# [Retracted] Isoquercitrin inhibits the progression of pancreatic cancer in vivo and in vitro by regulating opioid receptors and the mitogen‑activated protein kinase signalling pathway

**DOI:** 10.3892/or.2023.8505

**Published:** 2023-02-20

**Authors:** Quan Chen, Ping Li, Yong Xu, Yang Li, Bo Tang

Oncol Rep 33: 840–848, 2015; DOI: 10.3892/or.2014.3626

Subsequent to the publication of the above article, a concerned reader drew to our attention that the flow cytometric plots shown in Fig. 2 had previously appeared in another article published in the journal *Oncology Reports* [Huang G, Tang B, Tang K, Dong X, Deng J, Liao L, Liao Z, Yang H and He S: Isoquercitrin inhibits the progression of liver cancer *in vivo* and *in vitro* via the MAPK signalling pathway. Oncol Rep 31: 2377–2384, 2014], suggesting that data purportedly showing results obtained under different experimental conditions had been derived from the same original source.

Given the errors that were identified in the compilation of Fig. 2 in this article, and the fact that all the data in this figure had been re-used from a previously published source, the Editor of *Oncology Reports* has decided that this article should be retracted from the publication owing to a lack of overall confidence in the presented data. The authors were asked for an explanation to account for these concerns, but the Editorial Office did not receive a satisfactory reply. The Editor apologizes to the readership for any inconvenience that might result from the retraction of this article.

